# Advancing CAR-T Therapy for Solid Tumors: From Barriers to Clinical Progress

**DOI:** 10.3390/biom15101407

**Published:** 2025-10-02

**Authors:** Sergei Smirnov, Yuriy Zaritsky, Sergey Silonov, Anastasia Gavrilova, Alexander Fonin

**Affiliations:** Institute of Cytology of the Russian Academy of Sciences, Tikhoretsky Ave. 4, St. Petersburg 194064, Russia

**Keywords:** CAR-T, cells, solid, tumor, clinical, efficacy next, generation, T-cells, immunotherapy

## Abstract

Therapy with chimeric antigen receptor (CAR)-T cells has revolutionized the treatment of hematological malignancies. However, their application in solid tumors remains a formidable challenge due to obstacles such as the immunosuppressive tumor microenvironment, tumor heterogeneity, and limited T cell persistence. Although second- and third-generation CAR-T cells have shown restricted efficacy in clinical trials, next-generation strategies—including cytokine-armored CAR-T cells (e.g., IL-15, IL-7/CCL19), logic-gated systems, and localized delivery approaches—demonstrate promising potential to overcome these limitations. This review examines the major barriers impeding CAR-T cell efficacy in solid tumors, evaluates clinical outcomes from conventional CAR constructs, and highlights innovative strategies being tested in recent clinical trials. Key advances discussed include the use of dominant-negative receptors (e.g., TGFβRII) to combat immunosuppression and the co-expression of bispecific T cell engagers (BiTEs) to address antigen escape.

## 1. Introduction

Alongside conventional modalities for treating solid tumors—such as surgery, radiotherapy, and chemotherapy—targeted immunotherapeutic strategies are gaining increasing prominence. The adoptive transfer of tumor-infiltrating lymphocytes (TILs) has received FDA approval for certain malignancies [1], and numerous cancer vaccines encoding tumor-associated antigens (TAAs) or tumor-specific antigens (TSAs) are under clinical evaluation [2]. In parallel, cell-based immunotherapies employing chimeric antigen receptor (CAR)-T [3] and CAR-natural killer (NK) [4] cells are being actively investigated for solid tumors. More recently, CAR-macrophages have emerged as a nascent therapeutic avenue, leveraging their enhanced capacity to infiltrate tumor sites [5]. Additionally, bispecific antibodies have shown considerable promise [6,7]. To understand why CAR-T cells offer distinct advantages, it is important to grasp their fundamental design. Unlike bispecific antibodies, which act as external “bridges” to force a temporary connection between a T cell and a cancer cell, CAR-T cells are living drugs. They are a patient’s own T cells that have been genetically engineered to permanently express a synthetic receptor—the CAR. This receptor combines the cancer-targeting ability of an antibody with the powerful killing machinery of a T cell. This integration allows for more precise control. For instance, CAR-T cells offer finer control over T-cell activation thresholds in response to antigen density [8]. This means they can be designed to respond only when they encounter a high density of cancer markers, potentially sparing healthy cells that express low levels of the same marker. Furthermore, CAR-T cells can be equipped with safety switches, such as inducible caspase systems, allowing clinicians to eliminate them if severe side effects occur [9]. They can even be controlled with light using optogenetic tools for spatial regulation [10]. Perhaps most importantly, they can be engineered with sophisticated “logic gates.” For example, an “AND-gate” CAR-T cell requires the presence of two different cancer markers to become fully activated, greatly reducing the risk of attacking healthy tissues that might express only one of those markers [11].

**Figure 1:**
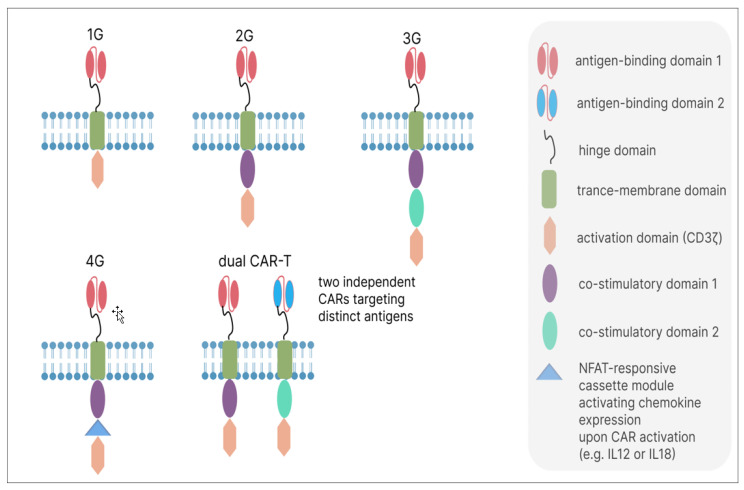
Generations of CARs. This figure illustrates the structural evolution of CAR-T cells across four generations. First-generation CARs (1G) consist of an extracellular single-chain variable fragment (scFv) for antigen binding, connected via a hinge and transmembrane domain to the intracellular CD3ζ signaling domain. Second-generation CARs (2G) add one co-stimulatory domain (e.g., CD28 or 4-1BB) to enhance T-cell activation and persistence. Third-generation CARs (3G) incorporate two co-stimulatory domains in series with CD3ζ to further boost signaling. Fourth-generation “armored” CARs (4G), often built on a 2G backbone, include an additional genetic element (e.g., a cytokine gene like IL-15) that is expressed upon T-cell activation to modify the tumor microenvironment. Dual CARs (dual CAR-T) are T cells that express two independent CARs targeting distinct antigens.

The evolution of CAR-T cells began with first-generation constructs (Figure 1). These comprised an extracellular antigen-binding domain (usually derived from an antibody) linked via hinge and transmembrane regions to the intracellular CD3ζ signaling module, which is essential for initiating T-cell activation [12]. However, these early CAR-T cells often failed to persist or function effectively in vivo. Subsequent incorporation of co-stimulatory domains—such as those from 4-1BB or CD28—gave rise to second-generation (2G) CARs. These additional signals act like a “second key” that the T cell needs to turn on fully, markedly improving its persistence, expansion, and effector functions in vivo [13]. Further refinements led to third-generation (3G) CARs, which combine multiple co-stimulatory signals (e.g., CD28 plus 4-1BB) in an attempt to further amplify anti-tumor efficacy [14]. Fourth-generation (4G) “armored” CAR-T cells are designed to deliver additional therapeutic payloads—such as cytokines—upon activation. These constructs typically build upon 2G architectures and incorporate transgenic cytokines under constitutive or nuclear factor of activated T-cells (NFAT)-inducible promoters [15,16,17]. This enables them to modify the local tumor microenvironment by secreting immune-stimulating molecules right where they are needed, for example. Dual-targeting CAR-T cells represent another advanced platform, wherein T cells express two independent CARs targeting distinct antigens. This approach can mitigate antigen escape through OR-gating logic (where engagement of either antigen activates the cell) or AND-gating logic (requiring both antigens for activation), the latter enhancing tumor specificity and reducing off-tumor toxicity [18,19,20]. Such designs fall under the broader paradigm of “logic-gated” CAR therapies [11]. Genetic engineering efforts now routinely extend beyond CAR insertion to include knockout (KO) of inhibitory genes—e.g., CD5 or NR4A transcription factors—using CRISPR-Cas9, further enhancing the proliferative capacity and anti-tumor potency of CAR-T cells [21,22]. In this review, we explore the obstacles that have limited the success of 2G and 3G CAR-T cells in solid tumors and highlight promising next-generation engineering strategies that have demonstrated potential in early-phase clinical trials.

## 2. Barriers to Effective CAR-T Cell Therapy in Solid Tumors

**Figure 2:**
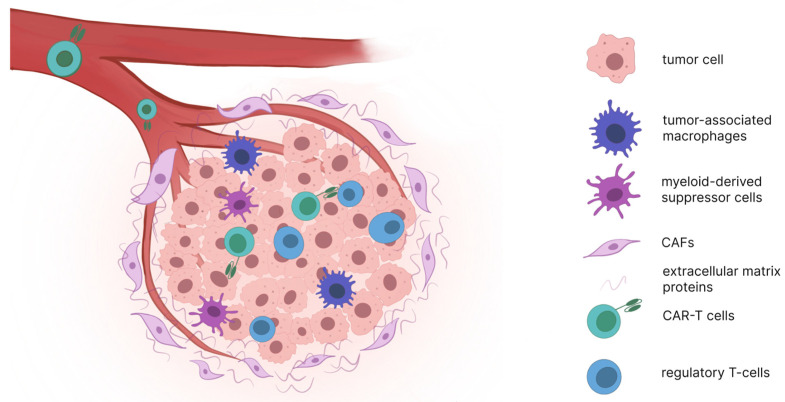
Solid tumor microenvironment. This figure depicts the multifaceted barriers within the TME. It includes the dense extracellular matrix (ECM) produced by cancer-associated fibroblasts (CAFs), the abnormal and leaky tumor vasculature, and the diverse population of immunosuppressive cells (regulatory T-cells, myeloid-derived suppressor cells, tumor-associated macrophages).

Factors limiting the efficacy of CAR-T therapy for solid tumors have been described in detail previously [23]. Unlike hematological cancers, successful targeting of solid tumors requires CAR-T cells to overcome a physical barrier composed of a dense layer of extracellular matrix proteins (Figure 2). This layer is formed by connective tissue cells, particularly cancer-associated fibroblasts (CAFs), which are activated under the influence of the signaling protein TGF-β (transforming growth factor beta) [24]. TGF-β, functioning as a chemokine, can also directly limit T-cell infiltration into solid tumors by inhibiting the expression of chemokine receptors such as CXCR3 on T-cells [25]. Preclinical studies have demonstrated that CAFs inhibit CAR-T cell function through complex mechanisms involving both TGF-β signaling and intercellular contact-mediated suppression [26]. The study by Li et al. [27] provides a compelling example of how to overcome this barrier. They engineered CAR-T cells to target two antigens (EGFR and IL13Rα2) while also expressing a dominant-negative TGF-β receptor (dnTGFβRII). A dominant-negative receptor is a mutated form that can bind TGF-β but cannot transmit its immunosuppressive signal into the cell, effectively acting as a decoy. By blocking this key immunosuppressive pathway, these “armored” CAR-T cells demonstrated significantly superior proliferation and anti-tumor activity in vivo compared to their non-armored counterparts. This highlights a direct strategy to neutralize a major component of the TME.

Another critical physical barrier leading to T-cell exclusion from the tumor microenvironment (TME) is the aberrant vascular network characteristic of solid tumors, which promotes tissue hypoxia and limits T-cell extravasation into the TME. Hypoxic conditions facilitate the recruitment of immunosuppressive cells through chemokine secretion and enhance the expression of immune checkpoint molecules including cytotoxic T-lymphocyte-associated protein 4 (CTLA4) and lymphocyte-activation gene 3 (LAG3) on regulatory T-cells (Tregs), as well as programmed cell death 1 ligand 1 (PD-L1) on myeloid-derived suppressor cells (MDSCs), tumor-associated macrophages (TAMs), and tumor cells [28]. The disordered vasculature further contributes to impaired T-cell infiltration by downregulating essential adhesion molecules such as vascular cell adhesion protein 1 (VCAM1) and intercellular adhesion molecule 1 (ICAM1) [29]. TAMs represent the most abundant immune population within the TME and suppress T-cell-mediated anti-tumor immunity through multiple mechanisms, including secretion of immunosuppressive cytokines like IL-10, production of amino acid-catabolizing enzymes such as arginase 1, and expansion of Treg populations [30].

Beyond physical and immunosuppressive barriers, the TME presents a profound metabolic challenge for CAR-T cells. Tumor cells exhibit a highly glycolytic metabolism, even in the presence of oxygen (the Warburg effect). This leads to the accumulation of high levels of lactate and protons, creating an acidic microenvironment. Lactate is not merely a waste product; it directly inhibits T-cell function by suppressing cytokine production (e.g., IFNγ, TNFα) and cytotoxic activity, and by promoting T-cell exhaustion [31]. Furthermore, the TME is often depleted of critical nutrients like glucose and glutamine, which are voraciously consumed by tumor cells, leaving CAR-T cells starved and unable to generate the energy needed for proliferation and effector functions. Another key immunosuppressive metabolite is adenosine. It is generated in the TME from extracellular ATP by the ectoenzymes CD39 and CD73, which are frequently overexpressed on tumor and stromal cells. Adenosine binds to the A2A receptor on T cells, triggering potent immunosuppressive signals that inhibit T-cell activation, proliferation, and cytotoxicity [32]. To combat this, researchers are developing strategies to metabolically “reprogram” CAR-T cells. One approach is to engineer them to overexpress metabolic enzymes that confer a fitness advantage. For example, expressing a mutant, constitutively active form of phosphoenolpyruvate carboxykinase 1 (PCK1) can enhance T-cell mitochondrial metabolism and improve their anti-tumor function in low-glucose conditions [33]. Another strategy is to knock out or inhibit the adenosine A2A receptor (A2AR) in CAR-T cells, making them resistant to adenosine-mediated suppression [34]. Alternatively, CAR-T cells can be engineered to secrete enzymes like CD39/CD73-blocking antibodies or adenosine deaminase to degrade adenosine directly within the TME [35]. These metabolic engineering approaches aim to equip CAR-T cells with the resilience to survive and function in the nutrient-poor, metabolically hostile landscape of solid tumors.

An additional challenge involves the limited trafficking of systemically administered CAR-T cells to tumor sites. Memory T-cells, which typically constitute the majority of infused cells, preferentially migrate to lymphoid organs or bone marrow due to their expression of homing receptors CCR7 and CD62L [36]. As noted by Albelda et al. [37], the strategy of using single infusions of long-persisting, less differentiated CAR-T cells (memory phenotype), which has proven successful for hematological malignancies, might be less effective for solid tumors. Instead, multiple infusions of shorter-lived but more aggressive effector cells might be preferable. This concept is supported by work from Textor et al., demonstrating superior anti-tumor activity against neuroblastoma and carcinoma xenografts by CAR-T cells incorporating a CD28 co-stimulatory domain (characterized by stronger activation signals and effector differentiation) compared to those with a 4-1BB domain (associated with longer persistence but more gradual activation) [38]. This approach is further corroborated by findings from Kantari-Mimoum et al., showing that CAR-T cell tumor infiltration depends on IFNγ-mediated upregulation of intercellular adhesion molecule 1 (ICAM-1) on tumor cells [39]. It appears that upon initial antigen encounter, effector-polarized CAR-T cells, particularly those with CD28 co-stimulation, produce substantial IFNγ, which enhances ICAM-1 expression on tumor cells and facilitates subsequent CAR-T cell infiltration into tumor tissue. Given that only a small fraction of administered CAR-T cells initially reach the tumor [40], robust IFNγ production likely plays a decisive role in their subsequent expansion and overall therapeutic efficacy.

## 3. Therapeutic Outcomes of 2nd and 3rd Generation CAR-T Cells in Solid Tumors

Recent clinical trial data indicate that conventional second-generation (2G) and third-generation (3G) CAR-T cells remain insufficiently equipped to address the challenges posed by solid tumors. Specifically, limitations in infiltration, persistence, and effector function have prevented achieving durable therapeutic responses with CAR-T cells targeting mesothelin [41], EGFRvIII [42,43,44], HER2 [45], and GD2 [46,47]. The outcomes of clinical trials targeting these and other antigens provide valuable insights into the factors restricting CAR-T cell efficacy.

### 3.1. Off-Tumor Antigen-Specific Activation

While previous sections addressed multiple factors limiting CAR-T cell efficacy in solid tumors, a fundamental constraint remains the scarcity of truly unique targets expressed exclusively on cancer cells and completely absent from healthy tissues [48]. Most targets for solid tumor CAR-T therapy are tumor-associated antigens (TAAs) that exhibit some expression in non-malignant tissues. Clinical trials targeting mesothelin (MSLN) illustrate this challenge of off-tumor toxicity [49]. MSLN is a surface protein associated with apoptosis resistance, invasion, and metastasis in various tumors including mesothelioma, pancreatic cancer, ovarian cancer, and lung cancer [50]. In clinical trials, dose-escalation of MSLN-targeted CAR-T cells led to severe pulmonary toxicity in patients with solid tumors receiving high T-cell doses (1–3 × 10^8^ cells per m^2^) [49]. This toxicity was attributed to MSLN expression by benign lung epithelial cells in affected lungs, with autopsies revealing acute lung injury accompanied by extensive T-cell infiltration and CAR-T cell accumulation in pulmonary tissue. Similar on-target, off-tumor toxicity was observed with CAR-T cells targeting human epidermal growth factor receptor 2 (HER2), including a fatal case of pulmonary toxicity in a patient with metastatic colon cancer resulting from low-level HER2 expression in healthy lung tissue [51]. Numerous additional cases of such toxicity in CAR-T clinical trials have been comprehensively reviewed by Hou et al. [52].

### 3.2. Heterogeneity in Antigen Expression

Even when tumor-specific antigens are identified, CAR-T cell therapy faces the challenge of antigen expression heterogeneity, a hallmark of solid tumors. The mutant EGFRvIII protein, resulting from exons 2–7 deletion in the epidermal growth factor receptor (EGFR) gene, represents an illustrative example [53]. While an ideal therapeutic target due to its exclusive expression on tumor cells, clinical trials with EGFRvIII-targeted CAR-T cells in glioblastoma patients resulted in the emergence of antigen-negative (EGFRvIIInull) tumor cells and subsequent disease progression [42].

As previously noted, HER2-targeted CAR-T cells have demonstrated significant toxicity [51]. Recent results from a clinical trial (NCT00902044) evaluating second-generation HER2-targeted CAR-T cells (with CD28 co-stimulation) in 14 patients with advanced sarcoma revealed severe respiratory adverse events (grade 3–4) in two patients. One patient experienced grade 4 cytokine release syndrome (CRS) with respiratory failure and near-complete bronchial obstruction adjacent to metastatic tumors. While complete responses were observed in 3 patients (21%) and stable disease in 4 patients (29%), the remainder showed progressive disease [54]. Reduced HER2 expression was detected in tumor samples from two patients following CAR-T therapy, with complete loss of HER2 expression observed in a patient with relapsed osteosarcoma.

Claudin-18.2 (CLDN18.2) has emerged as a promising target for immunotherapy of gastric and pancreatic cancers. This protein exists as two splice variants: CLDN18.1 (expressed in lungs) and CLDN18.2 (expressed in stomach) [55]. Normal tissue expression of CLDN18.2 is restricted to short-lived differentiated epithelial cells of the gastric mucosa [56], while this isoform is expressed on primary gastric malignancies and their metastases, and is also detected in pancreatic, esophageal, ovarian, lung, and colitis-associated colorectal tumors. In the largest clinical trial to date (NCT03874897, n = 98), therapy with second-generation CLDN18.2-targeted CAR-T cells (containing CD28 co-stimulatory domain) achieved an overall response rate (ORR) of 38.8% and disease control rate (DCR) of 91.8% [57]. Grade 1–2 cytokine release syndrome (CRS) occurred in 96.9% of patients, with no grade ≥ 3 CRS events, treatment-related deaths, or other significant toxicities reported. However, median progression-free survival and overall survival were limited to 4.4 months and 8.8 months, respectively, with only one patient maintaining response at the time of publication. The authors attributed the transient response duration to heterogeneous CLDN18.2 expression. Off-tumor toxicity was reported in six patients, primarily featuring grade 1–2 gastric mucosal injury with one case of grade 3 mucosal erosion. The relatively higher ORR observed in CLDN18.2-targeted therapy compared to other studies may be partially explained by recent findings that CLDN18 promotes cytotoxic T-lymphocyte accumulation by facilitating direct T-cell-tumor cell interactions through mobilization of the adhesion protein ALCAM into tumor cell membrane lipid rafts [58].

A notable clinical case [59] involved a 72-year-old male with ductal adenocarcinoma of the pancreas who experienced disease relapse with multiple metastases (liver, abdominal cavity, cervical lymph nodes) following multiline chemotherapy. Treatment with second-generation CLDN18.2-targeted CAR-T cells (containing 4-1BB co-stimulatory domain) resulted in a complete response (CR) that was maintained for 8 months before relapse occurred due to emergence of CLDN18.2-negative tumor cells.

### 3.3. Limited Persistence and Exhaustion

Patients with solid tumors consistently demonstrate reduced CAR-T cell expansion and persistence compared to those with hematological malignancies. For example, CLDN18.2-targeted CAR-T cells showed a mean maximum concentration (Cmax) of 6713 copies/μg genomic DNA [57], substantially lower than the 58,570 copies/μg genomic DNA observed in responders with chronic lymphocytic leukemia treated with CD19-targeted CAR-T cells [60].

Similarly limited persistence was observed in treating H3K27M-mutant diffuse midline gliomas (DMG) expressing high levels of disialoganglioside GD2 (NCT04196413) [61]. Despite restricted GD2 expression in normal tissues (primarily central nervous system and peripheral sensory nerves) [62], second-generation GD2-targeted CAR-T cells (with 4-1BB co-stimulation) demonstrated acceptable safety at low dose levels [61]. Notably, patients with bulky thalamic or cerebellar disease were excluded due to increased toxicity risks observed in murine models [63], as were patients with clinically significant dysphagia indicating substantial medullary dysfunction. Eleven patients with H3K27M-mutant pontine glioma or spinal DMG received GD2-CAR-T cells at two dose levels (DL1: 1 × 10^6^/kg; DL2: 3 × 10^6^/kg) following lymphodepleting chemotherapy. Patients showing clinical improvement received additional intracerebroventricular (ICV) infusions (10–30 × 10^6^ GD2-CAR-T cells), with some receiving up to 16 infusions. Four patients achieved significant tumor volume reductions (52%, 54%, 91%, and 100%), with one patient maintaining a complete response for over 30 months from study inclusion until the time of publication.

Thus, unlike the enhanced persistence mediated by 4-1BB co-stimulation in hematological malignancies, CAR-T cells targeting GD2 with 4-1BB domains failed to achieve sustained persistence in patients with diffuse gliomas, necessitating repeated administrations. This highlights a key point: the rules learned from blood cancers do not always apply to solid tumors. The hostile tumor microenvironment (TME) of a glioma appears to overwhelm the intrinsic persistence advantage typically conferred by 4-1BB signaling—likely due to factors such as T-cell exhaustion, immunosuppressive cytokines, metabolic competition, and poor T-cell trafficking. To overcome this barrier, combination approaches may be essential to enhance the persistence and functionality of second-generation CAR-T cells. A compelling example comes from a clinical trial of CLDN6-specific CAR-T cells (NCT04503278) [64], in which an RNA-lipoplex vaccine encoding the target antigen CLDN6 was co-administered. This vaccine strategy significantly prolonged CAR-T cell persistence, exceeding 100 days in all lymphodepleted patients by actively engaging the endogenous immune system. Specifically, the vaccine facilitated antigen presentation by dendritic cells in secondary lymphoid organs [65], thereby re-stimulating CAR-T cells and promoting their expansion, functional rejuvenation, and long-term survival through continuous antigen exposure in an immunologically supportive milieu. Importantly, this “in vivo boosting” mimics physiological T-cell priming, counteracting the exhaustion and anergy often induced by the immunosuppressive solid TME.

The CLDN6 gene is completely silenced after fetal development and absent from healthy adult tissues [66,67], but shows frequent aberrant surface expression in various solid tumors, making it an ideal target. In this trial, enhanced CAR-T expansion was particularly noted in patients receiving the lower cell dose (DL1: 1 × 10^7^ cells) combined with vaccine—suggesting that vaccine-driven immune stimulation may reduce the threshold of CAR-T cells needed for clinical efficacy. Partial responses were observed in six patients, with one complete response. Recent updates from this study (Annals of Oncology 2024, 35 (Suppl. 2), S482–S535) report an overall response rate of 38% and disease control rate of 69%.

This demonstrates that combining CAR-T therapy with strategies to boost endogenous immunity, such as RNA vaccines that amplify antigen presentation and sustain T-cell activation can be a powerful approach to overcome the persistence barrier in solid tumors. Rather than relying solely on engineered co-stimulation domains, leveraging the body’s own immune infrastructure may be key to unlocking durable CAR-T responses where traditional approaches have faltered.

## 4. Results of Clinical Trials of Next-Generation CAR-T Cells

In the previous section, we addressed several barriers faced by CAR-T cell therapy in the context of solid cancers. When summarizing the results of clinical studies involving second- and third-generation CAR-T cells, it becomes evident that in the vast majority of patients, even those who initially responded to therapy, the disease ultimately progressed and led to fatal outcomes. These results strongly indicate the necessity to develop therapeutic agents with a more diversified spectrum of activity against tumors, capable of engaging multiple targets rather than single antigens, while simultaneously influencing the tumor microenvironment and counteracting immunosuppressive mechanisms. This review does not attempt to comprehensively list all strategies for creating next-generation CAR-T cells aimed at overcoming these barriers that are currently under development at preclinical stages, nor does it cover all initiated clinical trials. For such extensive overviews, we direct readers to existing detailed review articles [68,69,70,71]. Instead, we will focus on strategies that have advanced to clinical trials and have published data available regarding their efficacy (Table 1). It should be noted that direct comparisons of 2G and 4G CAR-T efficacy are inherently limited by heterogeneity across studies, including differences in patient cohorts, tumor burden, lymphodepletion regimens, dosing strategies, and trial design.

### 4.1. Strategies Aimed at Increasing Persistence, Infiltration, and Effector Functions

Recent clinical trials involving 27 children with neuroblastoma demonstrated that fourth-generation GD2-specific CAR-T cells incorporating a suicide cassette based on an inducible caspase system [72] achieved an overall response rate of 63 percent (17 out of 27 children), including 9 patients with complete response and 8 with partial response. The three-year overall survival rate among patients who received the recommended dose reached 60 percent.

The authors noted that relatively favorable persistence of GD2-CART01 cells exceeding six weeks correlated with treatment response and was potentially associated with the presence of two co-stimulatory domains (CD28 and 4-1BB) in the CAR structure, in addition to specific CAR-T cell cultivation conditions involving the presence of IL-7 and IL-15 in the culture medium [73].

This trial by Del Bufalo et al. [72] is a landmark study, demonstrating that durable remissions are possible in a solid tumor with next-generation CAR-T design. The use of dual co-stimulation (CD28 for strong initial activation and 4-1BB for persistence) combined with a cytokine-enriched manufacturing process appears to have created a highly potent product. The fact that antigen loss was not observed upon relapse suggests that other mechanisms, like the immunosuppressive TME, were the primary drivers of treatment failure, pointing to the next frontier for improvement. The authors also observed that disease severity at the time of infusion served as a predictive factor for treatment response. Notably, this study did not document loss of GD2 expression on relapsed tumor cells following cessation of treatment response. The authors suggest that other factors, particularly immunosuppressive mechanisms, were responsible for the reduction in anti-tumor activity observed in this study. In subsequent research, the same investigative team demonstrated that during therapy of neuroblastoma patients with GD2-targeted CAR-T cells, polymorphonuclear myeloid suppressor cells diminished their anti-tumor efficacy [74]. Specifically, an increase in polymorphonuclear myeloid suppressor cell numbers in peripheral blood was observed in patients after CAR-T cell treatment in cases of relapse and loss of treatment response. Co-cultivation of these cells with CAR-T cells resulted in downregulation of genes involved in CAR-T cell activation.

The efficacy of CAR-T cell therapy in patients with solid tumors remains substantially limited by the tumor microenvironment, which contains numerous inhibitory signals that block immune responses [75]. Additionally, the tumor microenvironment typically maintains insufficient concentrations of cytokines such as IL-15 that are necessary for the survival and optimal functioning of tumor-specific T-cells [76]. During the ATHENA clinical trial, investigators evaluated a CAR-T cell therapeutic agent targeting the GPC3 antigen (AZD5851) in patients with advanced or recurrent hepatocellular carcinoma [77]. The study compared efficacy and side effects in a homogeneous patient population receiving either second-generation CAR-T cells or fourth-generation CAR-T cells co-expressing IL-15 (15.CAR-T) while maintaining identical antigen-recognition and co-stimulatory domains (4-1BB). This direct, head-to-head comparison is exceptionally valuable. It provides clear, controlled evidence that arming CAR-T cells with IL-15 can overcome the functional impairment seen with conventional CAR-T cells in this setting. The transcriptomic data showing upregulation of cytolytic genes (GZMs, PRF1) and survival regulators (FOS/JUN family) in the 15.CAR-T cells offers a mechanistic explanation for their superior anti-tumor activity. GPC3, a member of the glypican family, represents a glycosylphosphatidylinositol-anchored proteoglycan located on the cell membrane of cancer cells that plays important roles in controlling cell division and regulating growth [78]. This antigen is expressed in various solid neoplasms, including hepatocellular carcinoma, which ranks as the third most common cause of cancer-related mortality worldwide [79], while remaining absent from healthy cells, making it an attractive immunotherapeutic target. The study enrolled 12 patients receiving second-generation CAR-T cells and 12 patients receiving fourth-generation CAR-T cells. All patients underwent lymphodepletion with cyclophosphamide and fludarabine prior to cell infusion. In the group receiving second-generation CAR-T cells, 6 patients received a dose of 1 × 10^7^ CAR-T cells per square meter (DL1), while 6 patients received 3 × 10^7^ cells per square meter (DL2). Both DL1 and DL2 infusions proved safe but did not generate any anti-tumor responses. Twelve patients in the other group received 15.CAR-T cells at a concentration of 3 × 10^7^ cells per square meter (DL2). The authors reported no significant difference in adverse events between patients receiving DL2 of conventional CAR-T cells (n = 6) and those administered 15.CAR-T cells (n = 12). However, infusion of 15.CAR-T cells increased the frequency of cytokine release syndrome, which was managed through IL-1/IL-6 blockade or activation of inducible caspase 9 (iC9) incorporated into the vector system, leading to apoptosis of CAR-T cells. Specifically, three patients received a single intravenous dose of rimiducid, a chemical inducer of iC9 dimerization, resulting in rapid symptom improvement, effective reduction in circulating 15.CAR-T cells, and normalization of inflammatory cytokine levels in all three cases. Among the six patients receiving second-generation CAR-T cells at DL2, no objective responses were observed, with three patients experiencing disease progression and three achieving stable disease. Of the 12 patients administered 15.CAR-T cells at the same dose, four showed disease progression, four achieved stable disease, and four exhibited partial response according to Response Evaluation Criteria in Solid Tumors (RECIST) [80]. Patients receiving 15.CAR-T cells demonstrated a disease control rate (incorporating both stable disease and progressive disease) of 66.7 percent (8 of 12 patients) and an objective response rate of 33.3 percent (4 of 12 patients). Transcriptomic analysis of CAR-T cell samples from peripheral blood revealed 3285 differentially expressed genes in 15.CAR-T cells compared to conventional CAR-T cells, including elevated expression of CD8A/B, ZNF683 (which encodes HOBIT), and genes associated with cytolytic activity such as GZMs, PRF1, and NKG7. The authors also isolated RNA from 15.CAR-T cells infiltrating tumors and compared gene expression patterns between patients who responded to therapy (demonstrating greater than 20 percent reduction in tumor size) and those who did not respond. Cells isolated from responding patients showed increased expression of genes associated with T-cell activation, type I interferon signaling, and activation of T-cell survival regulator genes including FOS, FOSB, JUN, JUNB, and JUND. The clinical efficacy of IL-15-armored GPC3-CAR-T cells (33.3% ORR vs. 0% in 2G controls) can be directly attributed to IL-15’s role in promoting T-cell stemness and metabolic fitness. Transcriptomic profiling revealed not only upregulation of cytolytic machinery (GZMB, PRF1) but also increased expression of anti-apoptotic (BCL-2) and memory-associated (TCF7, IL7R) genes in circulating CAR-T cells from responders. This suggests IL-15 ‘armoring’ counteracts the exhaustion program typically induced by the hepatocellular carcinoma microenvironment, a hypothesis further supported by the observation that non-responders exhibited higher baseline serum levels of TGF-β and adenosine, known suppressors of IL-15 signaling. Thus, IL-15 co-expression does not merely boost proliferation, it actively reprograms CAR-T cells to resist key immunosuppressive metabolites prevalent in advanced solid tumors. Notably, the lack of response in half of the patients receiving IL-15-armored CAR-T cells was not random—it correlated with distinct biological features.

The authors noted that despite enhanced differentiation toward effector cells, 15.CAR-T cells exhibited significantly greater expansion compared to second-generation CAR-T cells. Collectively, these findings demonstrate that IL-15 co-expression induces a mixed program of effector differentiation and increased proliferative capacity that correlates with improved anti-tumor activity.

Preclinical investigations have demonstrated that secretion of IL-7 and CCL19 promotes infiltration and survival of murine CAR-T cells in vivo [81,82,83]. A phase 1 clinical trial (NCT03198546) evaluating CAR-T cells with constitutive expression of IL-7 and CCL19 in six patients with progressive hepatocellular carcinoma, pancreatic carcinoma, and ovarian carcinoma expressing either GPC3 or MSLN showed promising results [84]. Researchers generated these CAR-T cells using double-sequential lentiviral transduction. The first lentiviral vector contained genes encoding a CAR with an antigen-recognition domain targeting either GPC3 or MSLN, while the second lentiviral vector contained genes encoding IL-7 and CCL19. In one patient with advanced hepatocellular carcinoma, treatment with anti-GPC3 CAR-T cells resulted in complete tumor disappearance within 30 days following intratumoral injection. In a patient with advanced pancreatic carcinoma, treatment with anti-MSLN CAR-T cells led to nearly complete tumor disappearance 240 days after intravenous infusion.

### 4.2. Blockade of Tumor Microenvironment Immunosuppression

C-CAR031 represents another next-generation CAR-T cell therapeutic agent targeting GPC3 that differs from the AZD5851 drug by expressing a dominant-negative transforming growth factor β receptor (dnTGFβRII) instead of additional IL-15 expression [85]. The rationale for targeting TGF-β is strong. The release of TGFβ from tumor or stromal cells plays a significant role in tumor development and immune evasion [86]. Research has established that elevated TGFβ levels in the tumor microenvironment represent one of the determining factors that inhibit T-cell infiltration into tumors and contribute to poor response to PD-1/PD-L1 inhibitor blockade [87,88]. The co-expressed dominant-negative TGFβ receptor lacks the intracellular domain required for downstream signaling, thereby effectively blocking TGFβ-mediated immunosuppression. Unlike wild-type TGFβ receptors that trigger SMAD-dependent immunosuppressive signaling upon ligand binding, the dominant-negative variant lacks the intracellular kinase domain—rendering it incapable of signal transduction. This strategy allows CAR-T cells to ‘soak up’ TGF-β in the microenvironment without succumbing to its inhibitory effects. The choice of dual targeting (EGFR + IL13Rα2) was deliberate: both antigens are frequently co-expressed in glioblastoma, reducing the likelihood of antigen escape—a common failure mode in single-target CAR-T therapies.

This molecular intervention translated directly into clinical benefit: 57% ORR in advanced HCC patients (NCT05155189) who had failed multiple prior therapies—a response rate unattainable with conventional 2G CAR-T in the same setting [85]. The striking 57% ORR achieved with dnTGFβRII-armored CAR-T cells in heavily pretreated HCC patients likely stems from direct neutralization of one of the TME’s most potent immunosuppressive axes. Unlike systemic TGF-β inhibitors, which often cause off-target toxicity, the dominant-negative receptor acts locally—only in CAR-T cells encountering tumor antigen. Post-treatment biopsies from responders showed a significant reduction in intratumoral Tregs and MDSCs, alongside increased CD8+/Treg ratios—confirming that TGF-β blockade restored immune cell infiltration and function. In contrast, non-responders exhibited persistent high expression of alternative immunosuppressive ligands (e.g., PD-L1, CD73), suggesting that while TGF-β is a master regulator, combinatorial targeting may be necessary for broader efficacy. Before receiving CAR-T cells, all patients had undergone an average of three to five lines of systemic therapy, with 83 percent of patients presenting with liver metastases. The median reduction in lesion size reached 42 percent.

Impressive results emerged from a clinical study of fourth-generation CAR-T cells designated BZDS1901, which target mesothelin and feature interferon-γ-activated secretion of an antibody blocking the PD-1 receptor [89]. Immunosuppression mediated through the PD-1/PD-L1 pathway is a major barrier in solid tumors. By engineering the CAR-T cells to secrete their own PD-1 blocker right at the tumor site, this strategy creates a localized, self-sustaining checkpoint blockade. This is a clever way to overcome the immunosuppressive TME without relying on systemic administration of checkpoint inhibitors, which can have broader side effects. The BZDS1901 drug was administered intravenously at doses ranging from 1 × 10^6^ to 2 × 10^7^ cells per kilogram, with a second infusion performed in the absence of disease progression. According to preliminary data from 11 patients with pleural mesothelioma expressing PD-L1, the objective response rate reached 63.6 percent, including one complete response lasting more than two years. Six patients achieved partial responses, while four patients maintained stable disease.

### 4.3. Strategy Aimed at Overcoming Heterogeneity

As mentioned in the previous section, clinical trials of second-generation CAR-T cells targeting EGFRvIII yielded disappointing results [42]. During these studies, no radiographic responses to therapy were observed, and relapsed tumor cells expressed wild-type EGFR protein while demonstrating substantial intratumoral infiltration by suppressive regulatory T-cells. The best outcome in this study was disease stabilization in one of ten patients with recurrent glioblastoma that persisted for 18 months of observation. The authors concluded that peripheral infusion of EGFRvIII-directed CAR T-cells mediated antigen loss and induced adaptive resistance in patients with recurrent glioblastoma. Subsequently, the same research team under the direction of Dr. Marcela V. Maus developed a bicistronic construct enabling expression of both a CAR specific for EGFRvIII with a 4-1BB co-stimulatory domain and a specific T-cell enhancer (BiTE) against EGFR, consisting of a single-chain variable fragment targeting EGFR connected via a linker to another single-chain variable fragment capable of binding CD3. EGFR represents an antigen frequently overexpressed in glioblastoma but also expressed in normal tissues [90].

Preclinical studies demonstrated that EGFRvIII-specific second-generation CAR-T cells failed to achieve complete regression of tumors with heterogeneous EGFRvIII expression, unlike next-generation CAR-T cells equipped with additional BiTE secretion capability (CART.BiTE). Furthermore, unlike EGFR-specific second-generation CAR-T cells, CART.BiTE cells did not cause toxicity toward human skin grafts in vivo [91]. The authors also documented the ability of secreted BiTEs to activate T-cells within the tumor microenvironment, including reprogramming regulatory T-cells into cytotoxic killers [92]. The CAR component, through co-stimulation mediated by 4-1BB signaling, helped reduce T-cell exhaustion resulting from activation via BiTE engagement. This combined approach led to increased proliferation and persistence of these engineered cells. Based on these findings, Dr. Maus’s team initiated the first phase 1 clinical study (NCT05660369) in patients with recurrent glioblastoma. Preliminary results from the first three patients have been published [93]. Treatment with CART.BiTE did not produce adverse events exceeding grade 3 or dose-limiting toxic effects. Within days following a single intraventricular infusion of CART.BiTE, sharp and rapid radiographic tumor regression occurred, although the response proved temporary in two of the three participants. Specifically, participant 2 received a single infusion of 10 × 10^6^ CART.BiTE cells via an intraventricular catheter. Magnetic resonance imaging performed on day 2 revealed an 18.5 percent reduction in tumor cross-sectional area, progressing to a 60.7 percent reduction by day 69 compared to baseline measurements before infusion. The therapeutic response persisted for 150 days, and at the time of publication, this patient remained in complete remission. The authors attributed tumor progression in two of the three participants to limited persistence of CART.BiTE cells, indicating the need for further refinement of this construct’s design.

The complete and rapid tumor regression observed in glioblastoma patients treated with EGFRvIII-BiTE CAR-T cells underscores the power of engaging the endogenous immune system. Secreted BiTEs not only recruit bystander T cells to eliminate antigen-negative tumor clones (overcoming heterogeneity) but also ‘re-educate’ the TME as evidenced by the conversion of immunosuppressive Tregs into IFNγ-producing cytotoxic effectors in patient CSF samples. This dual mechanism, direct tumor killing by CAR-T cells plus indirect killing and TME remodeling by BiTE-activated polyclonal T-cells creates a self-amplifying anti-tumor loop. The transient nature of responses in two of three patients, however, highlights that even this sophisticated design cannot fully overcome the glioma’s metabolic hostility (e.g., lactate, hypoxia) without additional metabolic engineering.

With local administration of CART.BiTE, the secreted BiTE molecules proved safe despite expression of their target antigen on healthy cells, which the authors attributed to predominant localization of CART.BiTE cells (with detectable BiTE on their surface) within the cerebrospinal fluid and minimal presence of such cells in peripheral blood (0–2 percent). These observations correlate with results obtained from animal models, which demonstrated localized BiTE secretion specifically at sites of contact with the cognate antigen [92].

### 4.4. Strategy Aimed at Overcoming Off-Tumor Antigen-Specific Activation

Carcinoembryonic antigen (CEA) expression becomes elevated in various malignant tumors, including colorectal cancer, medullary thyroid cancer, and breast cancer, among others [94]. Low-level CEA expression may also occur in normal colon tissues. CAR-T cell therapy directed against this antigen in colorectal cancer patients can cause severe refractory colitis [95]. Recent data from a phase I trial (NCT05396300) evaluated a CEA-CAR-T cell therapeutic agent designed to activate only under hypoxic conditions [96], thereby restricting cell activity primarily to tumor areas while sparing healthy tissues. This is an elegant example of using the TME’s own characteristics—in this case, hypoxia as a safety switch. Previous preclinical studies established that compared to conventional CAR-T cells, hypoxia-activated CAR-T cells maintained a less differentiated state and exhibited enhanced oxidative metabolism and proliferation during in vitro cultivation [97]. These cells also demonstrated an ability to mitigate the negative effects of hypoxia on T-cell proliferation and metabolism, while generating more sustained anti-tumor activity in vivo compared to their conventional counterparts. During clinical trials, CAR-T cells were administered via intraperitoneal or intravenous routes using dose escalation, including low dose (1–3 × 10^6^ cells per kilogram) and high dose (4–6 × 10^6^ cells per kilogram) regimens, to 40 patients with solid cancers (including 35 with colorectal cancer and 3 with gastric cancer) [96]. Grade 1–2 mucositis occurred in 25 percent of patients, while immune-mediated diarrhea and colitis of all grades affected 32.5 percent of patients, including 17.5 percent with grade 3 events. No treatment-related deaths were recorded. Intraperitoneal administration yielded a higher objective response rate of 25 percent and disease control rate of 88 percent, compared to 8 percent and 67 percent, respectively, in the intravenous administration group.

According to results from a clinical study of second-generation CEA-CAR-T cells (incorporating a CD28 co-stimulatory domain) in 10 colorectal cancer patients [98], despite using significantly higher doses (1 × 10^8^ cells per kilogram versus 4–6 × 10^6^ cells per kilogram), the best outcome achieved was disease stabilization in 7 of 10 patients. No partial responses or significant tumor mass reductions were documented. CAR-T cells were detected in peripheral blood and tumor tissues of patients receiving high doses for periods ranging from several days to several weeks. In the study of hypoxia-activated CAR-T cells [96], the high-dose group receiving intraperitoneal infusion showed an increased objective response rate of 28.5 percent, with one patient demonstrating 76 percent regression of target lesions over six months of observation. Although the authors did not provide specific data on CAR-T cell persistence, they noted high expansion levels even at low doses. Thus, implementation of a CAR-T cell activation regulation system, combined with local infusion approaches, facilitated treatment responses in some patients.

### 4.5. Local vs. Systemic Administration

The choice of administration route is a critical determinant of CAR-T cell therapy success in solid tumors. Systemic (intravenous, IV) administration is the standard route, leveraging the body’s circulatory system to deliver cells throughout the body. This is ideal for disseminated disease but faces significant hurdles in solid tumors: poor extravasation from blood vessels, inefficient homing to the tumor site, and dilution of the therapeutic dose. In contrast, local administration routes—including intratumoral (IT), intraperitoneal (IP), or intracerebroventricular (ICV)—deliver the CAR-T cells directly to the site of disease. This bypasses trafficking barriers, achieves high local concentrations, and can reduce systemic exposure and associated toxicities (e.g., the reduced colitis seen with IP CEA-CAR-T [95] compared to IV). The dramatic responses seen with local delivery in trials targeting GPC3 (IT) [85], GD2 (ICV) [61], and EGFRvIII (ICV) [93] underscore its potential. However, local delivery is often impractical for metastatic disease and can be invasive. The future likely lies in a combined approach: using systemic delivery for widespread disease, potentially enhanced with homing receptor engineering, while reserving local delivery for accessible, bulky, or sanctuary site tumors (like the brain).

### 4.6. Allogeneic (“Off-the-Shelf”) CAR-T Cell Therapies

All the clinical trials discussed so far have used autologous CAR-T cells, which are manufactured from the patient’s own T cells. While personalized, this process is time-consuming (weeks) and costly, and the quality of the starting T cells can be poor in heavily pre-treated patients. Allogeneic CAR-T cells, derived from healthy donors, offer a promising alternative as a readily available “off-the-shelf” product. The major challenge is graft-versus-host disease (GvHD) and host rejection of the donor cells. To overcome this, donor T cells are genetically edited (e.g., using CRISPR/Cas9 or TALENs) to knock out the endogenous T-cell receptor (TCR) to prevent GvHD and often the HLA class I molecules to reduce host rejection [99]. While allogeneic CAR-T therapies have shown success in hematologic malignancies (e.g., ALLO-501 for lymphoma), their application in solid tumors is still in early clinical development. Key questions remain about their persistence and efficacy in the harsh solid TME compared to autologous cells. Importantly, the “off-the-shelf” nature of allogeneic CAR-T products may uniquely align with the emerging therapeutic paradigm for solid tumors, as proposed by Albelda et al. [37]. Unlike hematologic malignancies, where long-persisting, memory-like CAR-T cells often correlate with durable remissions, solid tumors may require multiple infusions of shorter-lived, highly activated effector T cells to overcome the immunosuppressive TME and achieve deep tumor penetration [37]. Autologous CAR-T manufacturing—with its weeks-long production timeline and variable cell quality—is poorly suited for this strategy. In contrast, allogeneic CAR-T products, derived from healthy donors and cryopreserved in large batches, can be readily available for repeated, precisely timed infusions.

Moreover, the future of allogeneic CAR-T therapy lies not merely in replicating autologous 2G/3G constructs, but in engineering next-generation “armored” platforms from the outset. Allogeneic 4G CAR-T cells—incorporating cytokine secretion (e.g., IL-15, IL-7/CCL19), dominant-negative receptors (e.g., dnTGFβRII), or bispecific engagers (e.g., BiTEs)—hold immense promise. These enhancements can be genetically encoded into the donor T-cell backbone alongside TCR and HLA edits, creating a standardized, scalable, and functionally superior therapeutic product. For instance, an allogeneic CAR-T co-expressing IL-15 and a PD-1-blocking nanobody could be administered in multiple doses to sustain effector function and counteract checkpoint-mediated exhaustion within the TME—a strategy impractical with patient-derived cells. While clinical data in solid tumors remain early [100,101,102], the convergence of allogeneic manufacturing with next-generation engineering represents a powerful synergy that may finally unlock the full potential of CAR-T therapy for solid malignancies.

**Table 1:** Efficacy of conventional and next-generation CAR-T cells.

Tumor Type	Target Antigen	CAR Design (Conventional)	CAR Design (Next-Gen)	ORR (Conventional)	ORR (Next-Gen)	Key Engineering Mechanism Driving Improvement	References (2G → 4G)
Hepatocellular Carcinoma	GPC3	2G (CD3ζ + 4-1BB)	4G (IL-15 secretion)	0% (0/12)	33.3% (4/12)	IL-15 enhances proliferation, cytolytic function, survival	[77] → [77]
Hepatocellular Carcinoma	GPC3	2G (CD3ζ + 4-1BB)	4G (dnTGFβRII)	0% (0/12)	57% (14/24)	Dominant-negative TGFβRII blocks immunosuppression	[77] → [85]
Glioblastoma	EGFRvIII	2G (CD3ζ + 4-1BB)	4G (EGFR-BiTE secretion)	0% (0/10)	100% (3/3)	BiTE recruits endogenous T-cells against antigen escape	[42] → [93]
Neuroblastoma	GD2	2G (CD3ζ + CD28)	3G + cytokine culture (IL-7/15) + iCasp9	27% (3/11 partial responses)	63% (17/27)	Dual co-stimulation + cytokine conditioning + inducible caspase safety system	[103] → [72]
Colorectal Cancer	CEA	2G (CD3ζ + CD28)	4G (Hypoxia-activated)	0% (0/10)	25% (3/14) IP route	Hypoxia-switch restricts activation to tumor, reducing toxicity	[98] → [97]
Mesothelioma	Mesothelin	2G (toxic, no efficacy reported)	4G (PD-1 nanobody secretion)	—	63.6% (7/11)	Local PD-1 blockade reverses TME immunosuppression	[49] → [89]

## 5. Discussion

Based on the reviewed clinical studies, it can be concluded that therapy using second and third-generation CAR-T cells in patients with solid cancers, while demonstrating certain therapeutic outcomes, has largely failed to induce long-term responses or achieve complete cure, with the exception of isolated cases where complete remission was observed. The administration of high doses of CAR-T cells was frequently complicated by toxicity toward healthy tissues and cytokine release syndrome. One of the principal factors contributing to disease progression was tumor heterogeneity, including the loss of target antigen expression, as illustrated in the aforementioned cases targeting EGFRvIII and CLDN18.2.

Therapy proved most effective when directed against truly tumor-specific antigens such as EGFRvIII or CLDN6, or when CAR-T cell activation could be selectively triggered by cancer cells exhibiting higher expression levels of tumor-associated antigens like CLDN18.2 or GD-2 compared to normal tissues. It is noteworthy that in studies targeting both CLDN18.2 and GD-2, the authors utilized a 4-1BB co-stimulatory domain within the CAR structure, which is known to enhance persistence in hematological malignancies [104] but also establishes a higher activation threshold relative to CAR-T cells incorporating CD28-based co-stimulation [105]. In clinical investigations of GD2-targeted CAR-T cells featuring the 4-1BB domain [62], the short duration of therapeutic response may be attributed to antigen density falling below the required activation threshold. Conversely, the more gradual activation kinetics associated with 4-1BB signaling may have prevented severe cytokine release syndrome, while the higher activation threshold potentially enabled discrimination between tumor cells with high GD-2 expression and healthy neurons with lower expression levels. Furthermore, the same study demonstrated that limitations in chemotactic efficiency and persistence could be overcome through repeated local infusions of CAR-T cells [61]. Simultaneously, sustained persistence, influenced by both CAR design and ex vivo cultivation methods, emerged as a critical determinant of successful intravenous CAR-T cell therapy in neuroblastoma patients [72].

At present, objective comparison between the efficacy of conventional second and third-generation CAR-T cells and next-generation products remains challenging due to considerable heterogeneity in patient cohorts and the limited number of patients treated with advanced CAR-T constructs to date. A notable exception is the clinical trial involving 24 patients with advanced or recurrent hepatocellular carcinoma [77], which directly compared second and fourth-generation CAR-T cells within a single patient cohort. This study demonstrated clear superiority of fourth-generation “armored” CAR-T cells, with IL-15-expressing constructs achieving responses where second-generation products failed. The systematic research conducted by Dr. Marcela V. Maus’s group further enabled comparative assessment of EGFRvIII-directed second and fourth-generation CAR-T cells across sequential clinical studies in recurrent glioblastoma patients. The engineering of CAR-T cells to secrete bispecific T-cell engagers (BiTEs) effectively addressed antigen heterogeneity and demonstrated superior efficacy. Across all studies of next-generation CAR-T cells, partial and complete responses have been documented even within small patient cohorts. Although response rates remain substantially lower than those achieved in hematological malignancies, these findings indicate that next-generation CAR-T cell approaches are advancing solid tumor immunotherapy to a new level of therapeutic potential.

Despite encouraging advances, most clinically developed CAR-T strategies for solid tumors still target only one of the many barriers to efficacy. Durable responses will likely require simultaneous disruption of multiple resistance mechanisms—a challenge that demands integrated, multidimensional solutions. The future of CAR-T therapy in solid tumors, as envisioned by the authors and supported by emerging clinical results, rests on four integrated pillars.

First, universal “off-the-shelf” CAR-T products, derived from healthy donors, promise to overcome the delays and variability of autologous manufacturing. By enabling immediate, repeatable administration ideally timed to match the kinetic profile of potent but transient effector cells, they align with evolving paradigms of dynamic tumor control.

Second, next-generation CAR designs are shifting from single-function tools to multifunctional platforms. These engineered cells not only target tumors more precisely, using logic-based antigen recognition to minimize escape, but also actively reshape their microenvironment, enhancing persistence through cytokine support, improving tumor homing, and incorporating molecular safety controls for on-demand elimination if toxicity arises.

Third, CAR-T therapy is evolving beyond monotherapy, increasingly deployed as a core component of rational combination regimens. Its synergy with checkpoint inhibitors, cancer vaccines, oncolytic viruses, and stromal-modulating agents offers a means to overcome the immunosuppressive barriers that have long limited its efficacy in solid tumors.

Fourth, and perhaps most consequential—timing matters. Mounting clinical evidence suggests that CAR-T cells achieve their greatest impact when deployed earlier in the disease course, before tumors become too large or immunologically entrenched. Shifting CAR-T into first- or second-line settings may be key to unlocking durable remissions, rather than reserving it as a last resort.

## Data Availability

Not applicable.

## Abbreviations

The following abbreviations are used in this manuscript:

CAR	Chimeric Antigen Receptor
ORR	Overall Response Rate
SD	Stable disease
PR	Partial response
DCR	Disease Control Rate
